# Fabrication of Anti-Aging TiO_2_ Nanotubes on Biomedical Ti Alloys

**DOI:** 10.1371/journal.pone.0096213

**Published:** 2014-05-02

**Authors:** Azhang Hamlekhan, Arman Butt, Sweetu Patel, Dmitry Royhman, Christos Takoudis, Cortino Sukotjo, Judy Yuan, Gregory Jursich, Mathew T. Mathew, William Hendrickson, Amarjit Virdi, Tolou Shokuhfar

**Affiliations:** 1 Department of Mechanical Engineering–Engineering Mechanics, Multi-Scale Technologies Institute, Michigan Technological University, Houghton, Michigan, United States of America; 2 Department of Bioengineering, University of Illinois at Chicago, Chicago, Illinois, United States of America; 3 Department of Restorative Dentistry, College of Dentistry, University of Illinois at Chicago, Chicago, Illinois, United States of America; 4 Department of Chemical Engineering, University of Illinois at Chicago, Chicago, Illinois, United States of America; 5 Department of Orthopedics, Rush University Medical Center, Chicago, Illinois, United States of America; 6 Research Resources Center, University of Illinois at Chicago, Chicago, Illinois, United States of America; 7 Department of Anatomy and Cell Biology, Orthopedic Surgery, Rush University, Chicago, Illinois, United States of America; 8 Department of Mechanical and Industrial Engineering, University of Illinois at Chicago, Chicago, Illinois, United States of America; 9 Department of Physics, University of Illinois at Chicago, Chicago, Illinois, United States of America; University of Akron, United States of America

## Abstract

The primary objective of this study was to fabricate a TiO_2_ nanotubular surface, which could maintain hydrophilicity over time (resist aging). In order to achieve non-aging hydrophilic surfaces, anodization and annealing conditions were optimized. This is the first study to show that anodization and annealing condition affect the stability of surface hydrophilicity. Our results indicate that maintenance of hydrophilicity of the obtained TiO_2_ nanotubes was affected by anodization voltage and annealing temperature. Annealing sharply decreased the water contact angle (WCA) of the as-synthesized TiO_2_ nanotubular surface, which was correlated to improved hydrophilicity. TiO_2_ nanotubular surfaces are transformed to hydrophilic surfaces after annealing, regardless of annealing and anodization conditions; however, WCA measurements during aging demonstrate that surface hydrophilicity of non-anodized and 20 V anodized samples decreased after only 11 days of aging, while the 60 V anodized samples maintained their hydrophilicity over the same time period. The nanotubes obtained by 60 V anodization followed by 600 °C annealing maintained their hydrophilicity significantly longer than nanotubes which were obtained by 60 V anodization followed by 300 °C annealing.

## Introduction

Different categories of biomaterials have been employed to repair bone loss injuries. Among various biomaterials used in implant materials in orthopedics and dentistry [1], titanium and its alloys have recently attracted attention because of several advantages [2]. Compared with other biometals used as implants, titanium and its alloys provide biocompatibility in terms of low ion release [3], excellent corrosion resistance [4], great mechanical properties in terms of high hardness, low elastic modulus and low density [5]–[10]. The biocompatibility of titanium is a result of the presence of surface native oxide layer (TiO_2_; titania; passive film) of 2–5 nm thickness which is naturally formed as titanium is exposed to air. This native oxide layer protects the bulk material from corrosion [11] and makes it bioinert [12]. Despite their bioinertness, titanium implants are sometimes encapsulated by fibrous tissue *in vivo* and show lack of osseointegration which can lead to infection and implant failure [13]. In addition, low pH and presence of lipopolysaccharide in saliva enhances corrosion rate of titanium dental implants [14]. In order to develop bioactivity and osseointegration, various surface modifications have been performed including hydroxyapatite (HA) and calcium phosphate coatings [15]; however these coatings could be delaminated at their interface with Ti because of difference in mechanical moduli [16].

To improve implant viability, nanotechnology holds certain advantages via modification of implant surfaces [17]. Recently, anodization techniques have been employed for formation of TiO_2_ nanotubes on titanium surfaces [18]–[20]. The anodized nanotubular surface shows promise for biomedical application due to increased osteoblast cell adhesion and function [18]–[20], increased growth of hydroxyapatite [21], [22], and improved cellular behavior and tissue integration [23]. Compared with a flat surface (low surface roughness), a nanostructured surface provides more surface area for protein adsorption and as a result cellular interaction is enhanced [24]. Due to their integration with bulk substrate, TiO_2_ nanotubes do not suffer from delamination [25] and improve osseointegration of the implant.

Formation of TiO_2_ nanotubes on Ti substrate has been shown to decrease water contact angle (WCA) [26]. Reduction of WCA is significantly desirable because low WCA is correlated with enhanced bone cell interaction with the surface after implantation [27]. The initial interaction between implant surface and its physiological environment can play a key role in preventing implant failure. It is known that biomaterials are immediately covered with proteins from blood and interstitial fluids following exposure to physiologic environment [28]. When a biomaterial is exposed to *in vitro* or *in vivo* conditions, proteins that are present in the cell culture media or fluids of the body adsorb on its surface in less than 1 s. Then, adsorbed protein functional groups (ligands) bond with cell surface receptors (integrins) [29]. Specifically, fibronectin and vitronectin are absorbed on the surface and form an intermediate layer that promotes cell adhesion [30], [31]. Steele et al. showed that amount of protein adsorption is higher on hydrophilic surface compared with hydrophobic surface [32]. Other studies report that favorable response of cells is increased on hydrophilic surface compared with hydrophobic surface [33]–[36]. Surface modification using anodization has shown strong impact on improving hydrophilicity and cell interaction. Desirable cellular response of various cell lines is increased on nanotubular surfaces compared to flat machined surfaces. Such enhancement is due to an increase of surface area, which results in expansion of available area for cell-substrate interaction, surface energy, hydrophilicity, protein adsorption and consequently cell adhesion [5], [25], [37]–[39].

Crystallinity is another factor that affects cellular behavior [40]. Under most anodization conditions, as-formed TiO_2_ nanotube structure is amorphous after anodization. Annealing at 450 and 600 °C for 3 h leads to formation of crystalline phases of anatase and rutile respectively. The relative amount of anatase formation is higher for the samples anodized with a higher voltage compared to the samples anodized at a lower voltage [26]. Crystallization of as formed TiO_2_ nanotubes further enhances desirable response of cells. MC3T3-E1 preosteoblast activity and tendency to spread increases as nanotube structure changes from amorphous to pure anatase, and is maximized when pure anatase transforms to an anatase-rutile mixture. Cell proliferation increases with increasing annealing temperature and apatite mineralization and corrosion resistance is maximized on rutile structure [5], [41]; however, the highest amount of filopodia formation and extension occurs on anatase structure [5], [41].

Similar to other tissues of body, natural bone structure possesses nano, micro, and macro scale features. In the current study, hierarchical micro-nano scale topography of titania (TiO_2_) is formed on the substrate via acid-etching and anodization respectively, in order to mimic natural bone morphology. In addition, cell functions, such as cell adhesion and gene expression, are promoted on acid-etched surfaces [42] and micro-nano scale structure of titanium surface increases hydroxyapatite formation and protein adsorption [43]. Although it is expected that a roughened surface on titanium and its alloys provides long term mechanical interlocking ability, it has not been explored to the extent of polished surfaces.

Previous studies have shown that hydrophobic Ti surfaces become hydrophilic after anodization, and hydrophilicity is further increased as the anodized surface is annealed [44]. Interestingly, the nanotubular surface loses part of its hydrophilicity after aging in air over a period of three months [44]. Considering the correlation between WCA and cellular response on bone implants, the aim of this study was to design hydrophilic surfaces that are able to maintain their low WCA. We hypothesized that optimized annealing and anodization parameters would improve hydrophilicity and minimize aging effect. Our results suggest that surface structure affects hydrophilicity and aging effect. Consequently, it is expected that such surfaces will maintain protein adsorption, cell adhesion, and osseointegration benefits of TiO_2_ nanotubes even after aging.

## Methods and Materials

### Fabrication of TiO_2_ nanotubes

In this study Ti-6Al-4V alloy (Ti-V) disks (McMaster-Carr) with 15 mm diameter and 1 mm thickness were used. Disks were sandblasted with alumina powder (Trinity Tool Company) and acid-etched with a 1∶1 ratio of sulfuric acid (Fisher) and hydrogen peroxide (30% Fisher) for 2 h. Samples were then washed with DI-water, sonicated in acetone for 30 minutes and dried in air.

In order to perform anodization, samples were connected to a voltage source (Keithley 2400 SourceMeter) as the working electrode and copper rod was used as the counter electrode. Both electrodes were immersed in electrolyte mixture of ethylene glycol (Fisher), 0.3 wt% NH_4_F (Fisher) and 2 vol% DI water. In order to investigate the effect of anodization voltage on WCA, two groups of samples were anodized at 20 and 60 V at room temperature for 4 h and a third group was left non-anodized for control. Table 1 shows the categorization of samples into different groups.

**Table 1:** Breakdown of samples into different groups in order to determine the optimal fabrication conditions.

Step 1	Step 2	Step 3	Step 4
			Non-annealed #2
			Annealed at 300 C for 1.5h #2
			Annealed at 300 C for 3h #2
		Non-anodized #14	Annealed at 450 C for 1.5h #2
			Annealed at 450 C for 3h #2
			Annealed at 600 C for 1.5h #2
			Annealed at 600 C for 3h #2
			Non-annealed #2
			Annealed at 300 C for 1.5h #2
			Annealed at 300 C for 3h #2
Sandblasted #42	Acid-etched #42	20 V anodized #14	Annealed at 450 C for 1.5h #2
			Annealed at 450 C for 3h #2
			Annealed at 600 C for 1.5h #2
			Annealed at 600 C for 3h #2
			Non-annealed #2
			Annealed at 300 C for 1.5h #2
			Annealed at 300 C for 3h #2
		60 V anodized #14	Annealed at 450 C for 1.5h #2
			Annealed at 450 C for 3h #2
			Annealed at 600 C for 1.5h #2
			Annealed at 600 C for 3h #2

In order to verify effect of anodization condition, two groups of roughened samples were anodized at 20 and 60 V while a third group was not anodized to be used as control. Samples of each group were annealed at 300, 450 and 600 °C for 1.5 and 3 h to verify effect of annealing condition. Finally various tests were performed to characterize the surfaces. # is used to show number of prepared samples in each group.

In order to verify effect of anodization voltage on TiO_2_ nanotubes dimension, a separate group of samples were anodized at 20, 40, 60, 70 and 90 V for 1 h in the electrolyte with same composition mentioned above.

### Annealing Treatment

To investigate the effect of annealing temperature and duration on WCA, anodized and non-anodized samples were annealed at 300, 450 and 600 °C for 1.5 and 3 h durations. A temperature controller was used to set and maintain the furnace temperature. Prior to annealing, samples were cleaned for 30 seconds with DI-water followed by N_2_ gas drying. The samples were then loaded into the furnace for thermal oxidization at the determined temperatures in ambient air. After heat treatment, samples were slow-cooled by moving them out of the furnace tube slowly at 5 cm every 7 minutes. This prevents the generation of micro-cracks caused by abrupt temperature change on the samples' surface. Table 1 shows how samples were categorized into different groups.

### Field Emission Scanning Electron Microscopy (FESEM)

Structural characterization of the prepared TiO_2_ nanotubes was performed using a FESEM (JEOL JSM-6320F). The samples were mounted on an aluminum stub with double-sided conductive carbon tape for imaging.

In order to image dimension of TiO_2_ nanotubes and determine their aspect ratio, TiO_2_ nanotubes were removed from substrate by scratching. Detached bundles of nanotubes were placed on a double-sided conductive carbon tape and attached to an aluminum stub for imaging. Finally, ImageJ software was used to measure dimensions of nanotubes.

### Energy Dispersive X-Ray (EDX)

EDX analysis was performed using JEOL JSM-6320F detector. A non-anodized sample and scratched-off nanotubes from surface of an anodized sample placed on carbon tape were verified to evaluate effect of anodization on chemical composition. Noran Voyager EDX software of the instrument was used to determine weight percentage of present elements according to their energy lines.

### White Light Interferometry

Surface roughness tests were performed on all of the samples after annealing using white light interferometry (NewView 6300, Zygo Corporation, Middlefield, Connecticut, USA). This optical microscope gathers light from the test area of the sample. The objective divides light into 2 paths (one that shines onto the surface of the test material, and another into an internal reference surface). Surface irregularities on the test material cause the measurement wave-front to travel different distances than the reference wave front, which cause interference bands when they are combined. This interference is read by a photo-detector, which translates the interference wave-front into a 3D image.

### Fourier Transform Infrared (FTIR) Spectroscopy

Crystalline structure and surface composition of all annealed samples were studied using diffused reflectance FTIR (FTIR, Nicolet, Model #ADU9700221). Prior to conducting FTIR scans, each sample was left inside the FTIR chamber for 1 h per purging in order to reduce noise. FTIR spectrum was obtained with 1 cm^−1^ resolution and 512 scans.

### Water Contact Angle measurement

To perform WCA measurements sessile drop method was used as standard protocol using a Rame'-Hart NRL CA Goniometer (M#100-0, S#2067). A micro-syringe (Hamilton, 802RN) was used to place a 5 µL of DI-water droplet onto surfaces. Wettability of the samples were monitored by taking WCA measurements (i) after anodization, (ii) after annealing and (iii) after annealed samples were aged in air.

### Statistical Analysis

In order to determine the differences in surface hydrophilicity and average surface roughness of various surfaces of Ti-6Al-4V samples, one-way ANOVA test was used. Tukey HSD post hoc analysis was used for pair-wise comparisons within these groups. Statistical software (SPSS v. 20.0, SPSS Inc., Chicago, IL, USA) was used for descriptive and statistical analyses. For all analyses, p-values <0.05 was considered statistically significant.

## Results

**Figure 1:**
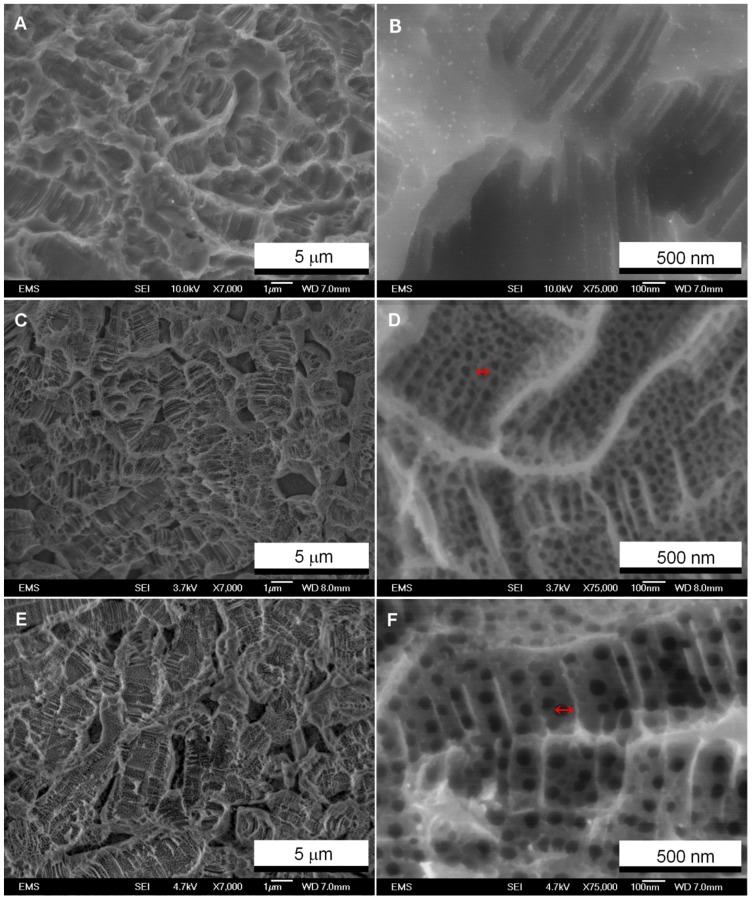
FESEM images of the surfaces. Images of non-anodized surface ((A) and (B)), 20 V anodized surface ((C) and (D)), and 60 V anodized surface ((E) and (F)) demonstrate formation of TiO2 nanotubes on anodized samples. Low magnification is shown at left side (A, C and E) and high magnification is shown on the right side (B, D and F).

The surfaces have been characterized by various techniques as explained above. The obtained results are as follows. FESEM images (Figure 1) show surface topography of non-anodized (Figure 1-A), 20 V anodized (Figure 1-C) and 60 V anodized (Figure 1-E) samples at low magnification. Low-magnification images of anodized surfaces (Figures 1-C and 1-E) show that vanadium-rich beta phase is dissolved. Dissolution of vanadium-rich phase of Ti-V during anodization in acidic electrolyte has previously been reported [45]. Presence of pores formed after dissolution, along with micro-scale roughness formed due to sandblasting and acid-etching provide a substrate that can establish long term mechanical bonding after implantation. Figure 1 also shows high-magnification images of surface topography for non-anodized (Figure 1-B), 20 V anodized (Figure 1-D) and 60 V anodized surfaces (Figure 1-F). It is observed that nanotubular structure is formed on anodized surfaces. Diameter of TiO_2_ nanotubes is about 100 and 50 nm on 60 and 20 V anodized sample respectively as marked by red arrows. These dimensions are compatible with previously reported dimensions of TiO_2_ nanotubes grown on the Ti substrate [46].

**Figure 2:**
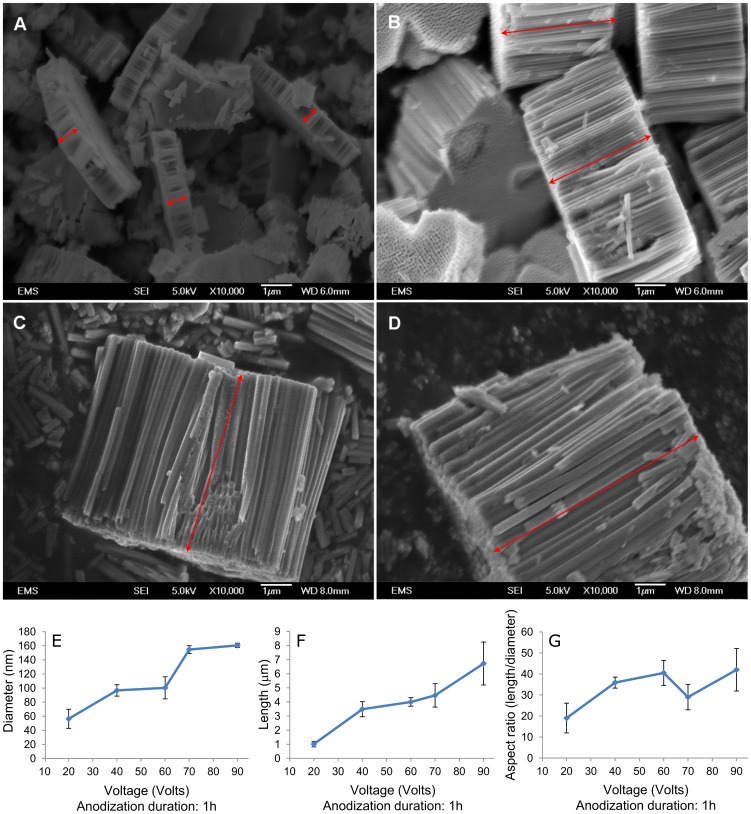
FESEM images show effect of anodization voltage on TiO_2_ nanotubes dimensions. Diameter and length of TiO_2_ nanotubes are increased as voltage is increased. (A) Shows 20 V anodized sample, (B) 40 V anodized sample, (C) 70 V anodized sample and (D) 90 V anodized sample. Effect of anodization voltage on TiO2 nanotubes dimensions is plotted in figures E–G. (E) Length, (F) diameter and (G) aspect ratio of TiO2 nanotubes are plotted versus anodization voltage. Length and diameter are enhanced as voltage is increased and aspect ratio seems to follow the same trend. Error bars show standard deviation for n = 3 samples.

FESEM images of TiO_2_ nanotubes anodized at different voltages are shown in (Figure 2A–D) for the 20 V anodized sample (Figure 2-A), 40 V anodized sample (Figure 2-B), 70 V anodized sample (Figure 2-C) and 90 V anodized sample (Figure 2-D). These images demonstrate length and diameter of TiO_2_ nanotubes increase as the anodization voltage is increased. As expected for nanotubes formed in organic electrolyte, TiO_2_ nanotubes are closely packed and have smooth walls without any perforation. Round bottom of nanotubes is observed at one side of detached layer while open top of nanotubes is noticed on the other side of nanotubular layer. Length and diameter of nanotubes were measured using ImageJ software and aspect ratio of tubes was calculated. At the bottom of Figure 2, the diameter of TiO_2_ nanotubes, length and aspect ratio are plotted versus voltage (Figure 2E–G). It can be observed that length and diameter are directly influenced by voltage and aspect ratio also seems to follow the same trend.

**Figure 3:**
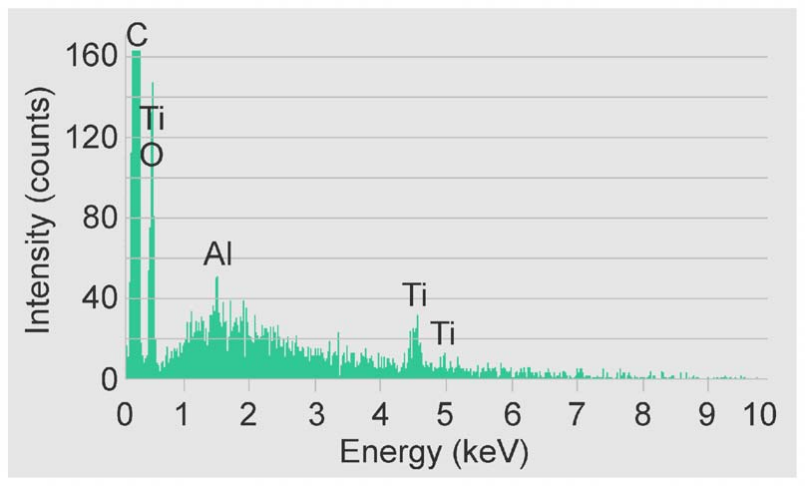
Energy Dispersive X-ray analysis. EDX confirms presence of titanium, oxygen and aluminum on the scratched-off nanotubes from surface of an anodized sample placed on carbon tape.

Using the EDX detector, chemical composition analysis was carried out on the surface of a non-anodized sample and scratched-off nanotubes from surface of an anodized sample placed on carbon tape (Figure 3). A peak indicating the presence of carbon can be seen; the carbon can be either residue of organic electrolyte decomposition or contamination from carbon tape. Comparison of chemical composition of surface before and after anodization suggests that vanadium is dissolved during anodization while oxide is formed (Table 2).

**Table 2:** Comparison of chemical composition of non-anodized surface and nanotubes.

Sample	Weight% of elements						
	Ti	Al	V	O	C	N	F
Non-anodized	87.8	5.1	4.6		1.3		
Scratched-off nanotubes placed on carbon tape	32	1.6	1.5	20	39.2	2.4	2.6

**Figure 4:**
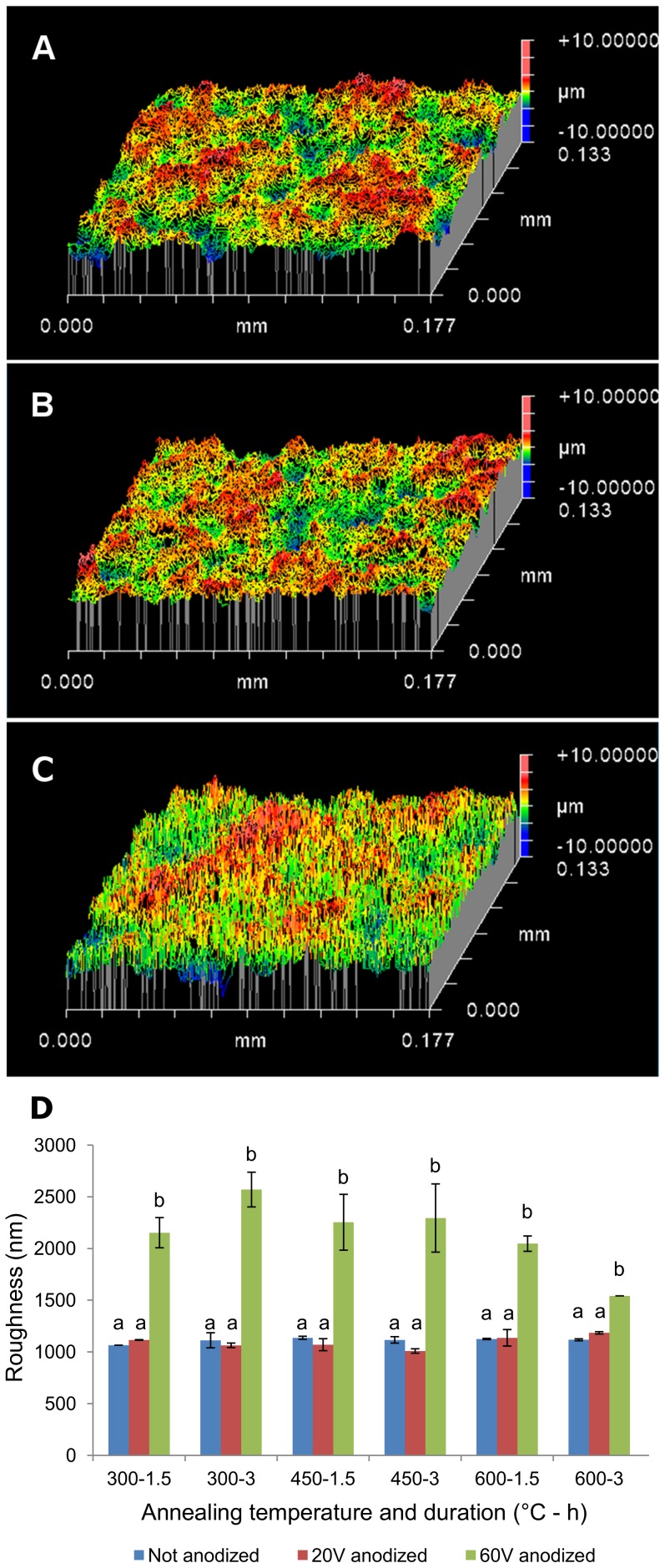
White light interferometry test of roughness. Roughness test performed on (A) non-anodized sample, annealed at 450 °C for 3 h, (B) 20 V anodized sample, annealed at 450 °C for 3 h, and (C) 60 V anodized sample, annealed at 450 °C for 3 h. 60 V anodized sample show different roughness compared to 20 V anodized sample and non-anodized sample. (D) White light interferometry test indicates 60 V anodized samples possess higher average roughness and higher root mean square roughness compared with 20 V anodized samples and non-anodized samples. Average roughness is related to surface area and consequently affects surface hydrophilicity. The x-axis indicates the temperature and duration of annealing [Temp (°C) - Time (h)]. a: *p<0.001*compared to b. Error bars show standard deviation for n = 2 samples.

Roughness measurements were performed after annealing samples (Figure 4). Roughness test results are illustrated for non-anodized samples annealed at 450 °C for 3 h (Figure 4-A), 20 V anodized sample annealed at 450 °C for 3 h (Figure 4-B) and 60 V anodized sample annealed at 450 °C for 3 h (Figure 4-C). Results of the roughness tests show that 60 V anodized samples have higher average roughness (Figure 4-D) and higher root mean square roughness (data not shown) compared with 20 V anodized samples. According to Wenzel's equation, enhancement of roughness is correlated with an increase of surface area, which results in enhanced hydrophilicity, protein adsorption, and cell-substrate interaction.

**Figure 5:**
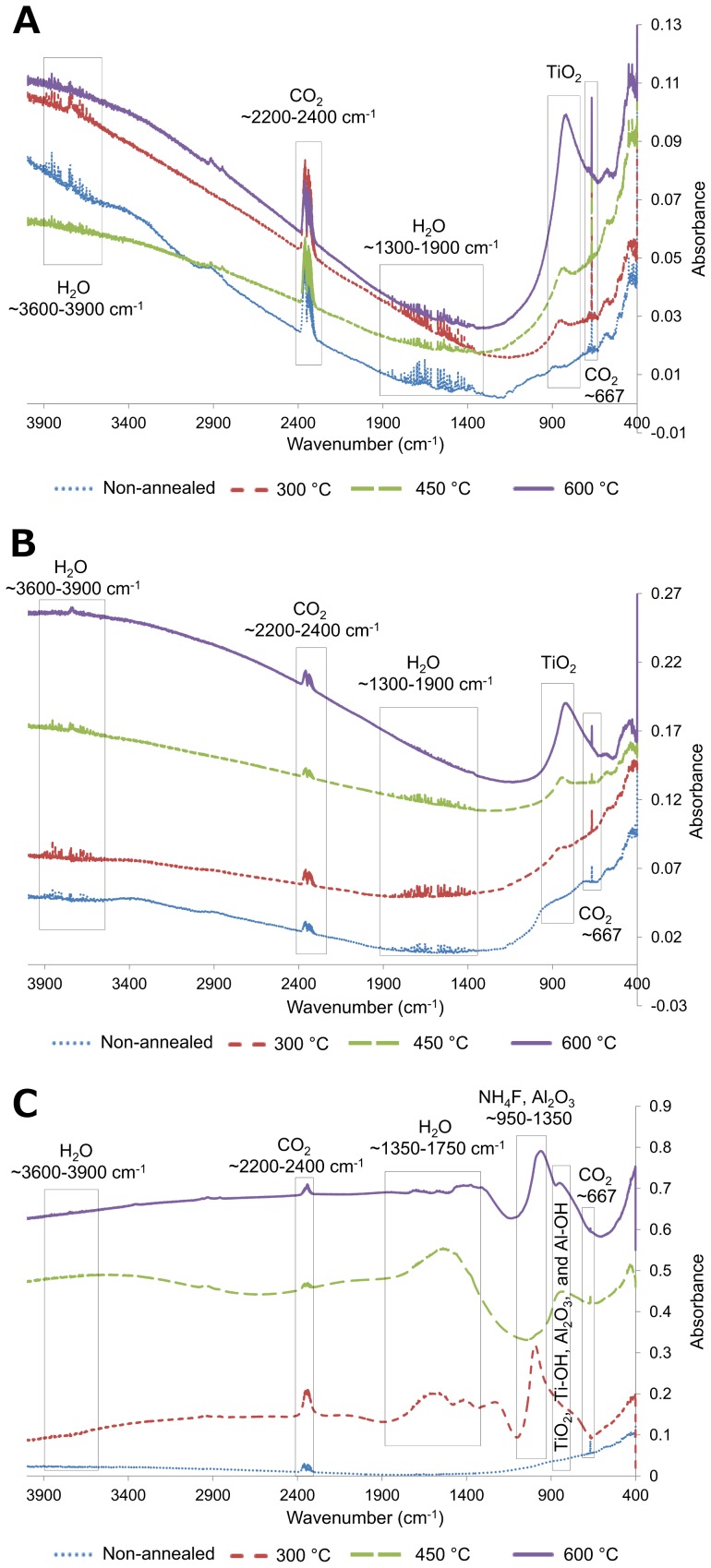
FTIR spectroscopy. FTIR spectra are shown for (A) non-anodized samples, (B) 20 V anodized samples and (C) 60 V anodized samples annealed at different temperatures for 3 h. The obtained spectra confirm the presence of TiO_2_ along with CO_2_ and H_2_O from the FTIR system. As annealing temperature is increased, the intensity of the TiO_2_ peak is enhanced. In addition, the TiO2 peak shifts from 898 cm^−1^ for non-annealed samples with amorphous structure to 870 cm^−1^ for annealed samples at 300 and 450 °C. Finally, the TiO_2_ peak shifts to 830 cm^−1^ for the samples annealed at 600 °C.

Figure 5 shows FTIR spectroscopy results for non-anodized (Figure 5-A), 20 V anodized (Figure 5-B) and 60 V anodized (Figure 5-C) samples, annealed at different temperatures for 3 h. Obtained spectra confirm the presence of TiO_2_ along with CO_2_ and H_2_O from the FTIR system. CO_2_ peaks are found at ∼2367 and ∼667 cm^−1^. H_2_O peaks are found at ∼3600–3900 and ∼1300–1900 cm^−1^. As annealing temperature is increased, the intensity of the TiO_2_ peak is enhanced. In addition, the TiO_2_ peak shifts from 898 cm^−1^ for non-annealed samples to 870 cm^−1^ for annealed samples at 300 and 450 °C. Finally, the TiO_2_ peak shifts to 830 cm^−1^ for the samples annealed at 600 °C. This is indicative of the presence of amorphous TiO_2_ on non-annealed samples, and formation of anatase structure TiO_2_ after annealing at 300 and 450 °C. Anatase TiO_2_, (870 cm^−1^) transforms into rutile TiO_2_, (830 cm^−1^) as annealing temperature is increased [47]. This behavior is observed for all samples regardless of anodization condition. For samples annealed at 300 and 450 °C, TiO_2_ with crystalline anatase structure is formed as well as rutile structure. For 60 V anodized samples (Figure 5-C), extra features are observed in the region 950–1350 cm^−1^ which represent Al-OH, Al-O-Al, V_2_O_5_ and Al_2_O_3_ [48]–[54].

**Figure 6:**
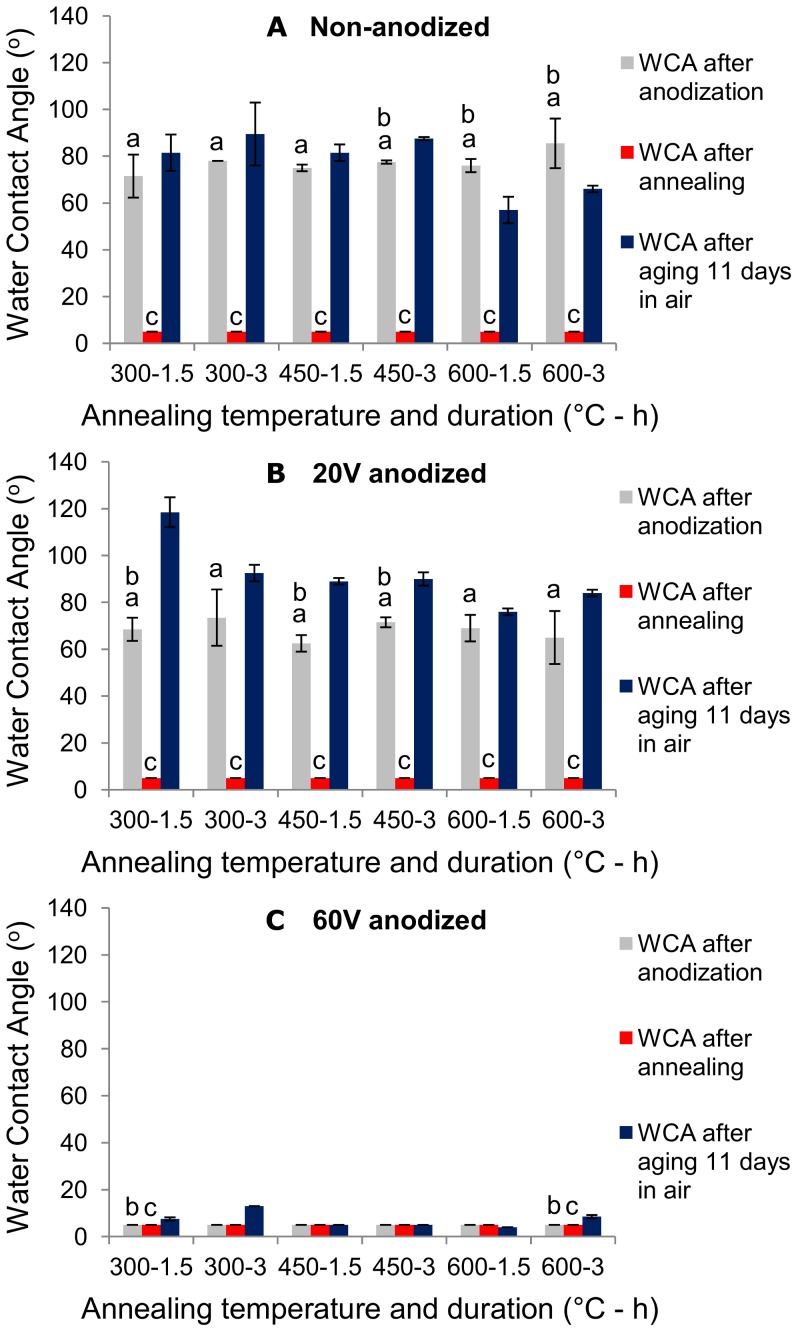
WCA measurements of the samples fabricated at different anodization and annealing conditions. WCA are shown for (A) not anodized samples, (B) 20 V anodized samples and (C) 60 V anodized samples. 60 V anodized samples possess low WCA after anodization and they are able to maintain their hydrophilicity when aged in air up to 11 days. The x-axis indicates the temperature and duration of annealing [Temp (°C)- Time (h)]. a: *p<0.05* compared to annealed group, b: *p<0.05* compared to aged group, c: *p<0.05* compared to aged group. Error bars show standard deviation for n = 2 samples.

Using WCA measurements, surface wettability behavior was investigated for anodized samples at various voltages along with different annealing temperatures and durations (Figure 6A–C). WCA of non-anodized (Figure 6-A) and 20 V anodized samples (Figure 6-B) were high, ranging from 50 to 120 °C. However, 60 V anodized samples show WCA below 5 °C (Figure 6-C). After annealing, all groups demonstrated WCA below 5 °C. WCA measurements of the samples after being aged in air for 11 days, show increase in WCA for non-anodized and 20 V anodized samples, ranging from 50 to 120 °C. However, 60 V anodized samples, maintained their low WCA after aging up to 11 days, regardless of annealing condition.

**Figure 7:**
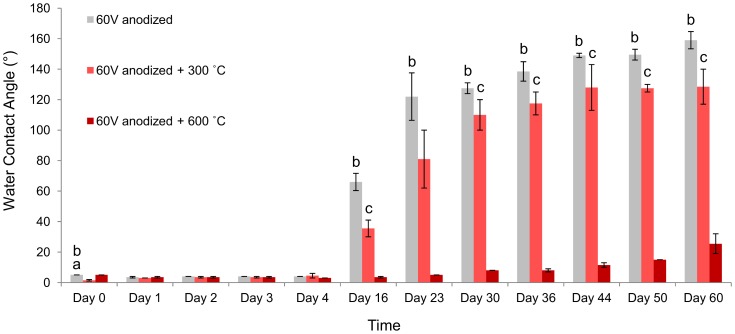
Effect of annealing temperature on aging behavior of 60 V anodized samples. Gray bars are non-anodized samples, light red bars are anodized samples that are then annealed at 300 °C for 3 h (60 V+300 °C) and dark red bars are anodized samples that are then annealed at 600 °C for 3 h (60 V+600 °C). 60 V anodized samples that are then annealed at 600 °C for 3 h, are significantly more able to maintain their hydrophilicity. a: *p<0.05* compared to 60 V+300 °C group, b: *p<0.05* compared to 60 V+600 °C group, c: *p<0.05* compared to 60 V+600 °C group. Error bars show standard deviation for n = 2 samples.

In order to verify effect of annealing temperature on aging behavior of TiO_2_ nanotubes, WCA measurements were performed up to 60 days. WCA measurements after prolonged aging indicate that annealing condition affects the ability of surface to maintain its hydrophilicity (Figure 7). As expected, 60 V anodized samples, annealed at 300°C for 3 h, are more able to maintain surface hydrophilicity compared with non-annealed samples. Interestingly, the 60 V anodized samples annealed at 600 °C for 3 h provide the highest ability in maintaining surface hydrophilicty. The samples that were prepared under optimum conditions (60 V anodized samples annealed at 600 °C for 3 h) provide stable, non-aging, hydrophilic surfaces, which maintain hydrophilicity for at least up to 60 days.

## Discussion

EDX results confirm the presence of titanium, vanadium and aluminum as components of Ti-V on non-anodized surface. However, scratched-off nanotubes show presence of oxygen; and relatively lower weight percentage of vanadium possibly due to the dissolution of vanadium during anodization (Table 2). The fluorine is hypothesized to be either residual or part of the chemical structure on the surface. The electrolyte is ammonium fluoride in ethylene glycol and water, and the fluorine is part of the chemical process of nanotube formation. The nitrogen may also be from the electrolyte. It should be noted that Ti is 44 g/mol and O_2_ is 32 g/mol, or oxygen weight% is 72% of the weight% of Ti. Therefore for the TiO_2_ nanotube values, the 20 weight% given for O is about 63% of the weight% for Ti, which indicates presence of oxygen equivalent to TiO_1.7_. In literature, the nanotube surface is a mixture of TiO_2_ and TiOH, which may explain the lower oxygen count. The Noran Voyager EDX software of the FESEM instrument was used to determine weight percentage of the elements according to their energy lines. The software fits a Kα+Kβ peak shape to the data to arrive at the K intensities. While Ti Kβ_1_ peak (4.93 keV) overlaps with V Kα_ 1,2_ (4.93 keV), the V Kβ_1_ peak (5.42 keV) has no overlap so it is possible to conclude V is decreased during anodization. Considering that the ratios of Kα to Kβ are very well known for transition metal K shells, the software is capable of deconvoluting Ti Kβ from V Kα. However, while the precision of oxygen quantification is questionable since Ti Lα (0.45 keV) and V Lα (0.51 keV) overlap the O Kα (0.52 keV), we are able to observe a relative increase in oxygen between the non-anodized and anodized samples.

Our results indicate that hydrophilicity after anodization is highly affected by applied anodization voltage. Before annealing, 60 V anodized samples show higher hydrophilicity compared to control (non-anodized) and 20 V anodized samples (Figure 6). Previous studies also show that surfaces of both Ti and Ti-V become hydrophilic after anodization and hydrophilicity is further increased as anodized surface in annealed [44], [55]. This behavior can be attributed to the increase in surface area after anodization, which results in enhancement of hydrophilicity, available area for adsorption, interaction, and consequently cell adhesion [5], [25], [37], [38]. Enhancement of roughness can generally lead to either the increase or decrease of water contact angle. In the case of formation of nanotubes on the surface of titanium dioxide, it has been widely reported that formation of nanotubes results in reduction of water contact angle [26], [44], due to presence of OH^–^ groups on the anodized surface in the form of Ti(OH)_4_ [44]. Therefore, it is assumed that air is not trapped underneath the liquid and Wenzel's equation can be applied for the TiO_2_ nanotubular surface. Based on the Wenzel relation, as surface roughness of a hydrophilic surface is enhanced, surface hydrophilicty is increased [56]. Our results, in compliance with previous studies [25], [26], show that surface roughness is increased after anodization (Figure 4). As anodization voltage increased, the surface area and nanotube size was also increased proportionally (Figure 1E–G). Thus, 60 V anodized samples show higher hydrophilicity compared to that of control (non-anodized) and 20 V anodized samples. In addition, application of higher voltage during anodization of both Ti and Ti-V materials can result in formation of as-anodizaed TiO_2_ nanotubes with some amount of crystalline structure [26] which, in turn, plays an important role in surface hydrophilicity. Higher anodization voltage also promotes formation of anatase crystalline structure obtained after heat treatment [55].

Our results indicate that annealing significantly decreases WCA (Figure 6). Nanotubular surfaces (WCA>50°) are transformed to superhydrophilic surfaces (WCA<5°) after annealing, regardless of annealing and anodization conditions. It seems surface structure - in terms of crystallinity and morphology - is the main influence on hydrophilicity, while electrolyte components present on the surface do not crucially affect WCA. When NH_4_F containing electrolyte is used for anodization, fluorine is present on the anodized surfaces. The fluorinated compound formed during anodization is water soluble and potentially affects hydrophilicity. Atsuta *et al*. reported hydrophilicity is improved after treatment with NH_4_F [57]; however, fluorine is thermally decomposed during annealing [5], [58]–[60] and cannot be responsible for hydrophilicity of annealed surfaces. Crystallinity and morphology are the factors that control surface hydrophilicity.

WCA measurements after aging (Figure 6) demonstrate that surface hydrophilicity of non-anodized and 20 V anodized samples decreases after 11 days of aging in air; however, annealed 60 V anodized samples maintain their hydrophilicity up to 11 days aging in air, regardless of annealing condition. The effect of annealing temperature demonstrated that the nanotubes that are obtained by 60 V anodization and annealing at 300 °C lose their hydrophilicity after 16 days of aging (Figure 7). Nevertheless, nanotubes that are obtained by 60 V anodization and annealing at 600 °C maintain their hydrophilicity even after 60 days of aging.

It is known that hydrophilicity is related to the presence of OH^–^ groups on the surface in the form of Ti(OH)_4_ after anodization. When anodized surface is aged in air, hydroxyl groups are transmitted to air to reach surface hydroxylation/dehydroxylation equilibrium. Since TiO_2_ is more stable than the Ti(OH)_4_, following reaction occurs: Ti(OH)_4_ → TiO_2_+2H_2_O [44]. Consequently, aged surfaces lose their hydrophilicity. Our data suggests that a relation exists between surface hydroxylation/dehydroxylation equilibrium and surface crystalline structure. Anatase crystalline structure is more potent in preventing the establishment of hydroxylation/dehydroxylation equilibrium compared with amorphous TiO_2_, while rutile crystalline structure provides the greatest resistance to the hydroxylation/dehydroxylation equilibrium process (Figure 5).

Dissolution of β phase of Ti-V along with micro-scale roughness of surface formed by sand-blasting would in turn assist mechanical bonding at bone implant interface. Optimized nano scale structure of nanotubes provides promising surfaces for implants used in dentistry and orthopedic applications.

## Conclusions

Hydrophilic nanotubular surfaces obtained by anodization improve cellular interaction. Considering the key role of surface hydrophilicity, we have studied optimization of anodization and annealing conditions. The following conclusions can be made from this study:

1. Our results indicate that the hydrophilicity of TiO_2_ nanotubes before annealing is highly affected by the anodization voltage. Before annealing, 60 V anodized samples show low WCA (<5°), while non-anodized and 20 V anodized samples show high WCA (>50°). Lower WCA of 60 V anodized samples can be due to presence of nanotubes with larger dimensions and higher surface roughness. However, the main factor that affects the maintenance and stability of the obtained hydrophilic TiO_2_ nanotubular surfaces is the annealing temperature.
2. Annealing significantly decreases the WCA (<5°). Nanotubular surfaces are transformed to hydrophilic surfaces after annealing, regardless of annealing and anodization conditions. However, WCA measurements after aging demonstrate that surface hydrophilicity of non-anodized and 20 V anodized samples decreases within 11 days of aging, while 60 V anodized samples maintain their hydrophilicity longer than 11 days. Anodization at high voltages partially promotes formation of crystalline structure which in turn enhances surface hydrophilicity.
3. The effect of annealing temperature demonstrated that nanotubular surfaces that are obtained by 60 V anodization and annealing at 300 °C lose their hydrophilicity after 16 days of aging. Nevertheless, nanotubeular surfaces that are obtained by 60 V anodization and annealing at 600 °C maintain their hydrophilicity up to 60 days. This behavior suggest that hydroxylation/dehydroxylation equilibrium on surface is established slower as amorphous structure is transformed to anatase crystalline structure and the slowest equilibrium process occurs when anatase is transformed to rutile.
4. Therefore, we conclude that in order to obtain nanotubes that do not age for several weeks in ambient conditions, an annealing temperature of 450 °C above is required.
